# Service providers’ experiences of disrespectful and abusive behavior towards women during facility based childbirth in Addis Ababa, Ethiopia

**DOI:** 10.1186/s12978-017-0449-4

**Published:** 2018-01-05

**Authors:** Anteneh Asefa, Delayehu Bekele, Alison Morgan, Michelle Kermode

**Affiliations:** 1 School of Public and Environmental Health, College of Medicine and Health Sciences, Hawassa University, P.O.Box 70, Hawassa, Ethiopia; 2 Nossal Institute for Global Health, School of Population and Global Health, The University of Melbourne, Level 5, 333 Exhibition Street, Melbourne, 3000 Australia; 3 Department of Obstetrics and Gynecology, Saint Paul’s Hospital Millennium Medical College, P.O.Box 143079, Addis Ababa, Ethiopia

**Keywords:** Disrespect and abuse, Mistreatment, Childbirth, Respectful maternity care, Service providers

## Abstract

**Background:**

Disrespect and abuse (D&A) of women during childbirth by the attending staff in health facilities has been widely reported in many countries. Although D&A in labor rooms is recognized as a deterrent to maternal health service utilization, approaches to defining, classifying, and measuring D&A are still at an early stage of development. This study aims to enhance understanding of service providers’ experiences of D&A during facility based childbirth in health facilities in Addis Ababa.

**Methods:**

A facility based cross-sectional study was conducted in August 2013 in one hospital and three health centers. A total of 57 health professionals who had assisted with childbirth during the study period completed a self-administered questionnaire. Service providers’ personal observations of mistreatment during childbirth and their perceptions of respectful maternity care (RMC) were assessed. Data were entered into and analyzed using SPSS version 16 software.

**Results:**

The majority (83.7%) of participants were aged <30 years (mean = 27.25 ± 5.45). Almost half (43.9%) were midwives, and 77.2% had less than five years experience as a health professional. Work load was reported to be very high by 31.6% of participants, and 28% rated their working environment as poor or very poor. Almost half (50.3%) of participants reported that service providers do not generally obtain women’s consent prior to procedures. One-quarter (25.9%) reported having ever witnessed physical abuse (physical force, slapping, or hitting) in their health facility. They also reported observing privacy violations (34.5%), and women being detained against their will (18%). Violations of women’s rights were self-reported by 14.5% of participants. More than half (57.1%) felt that they had been disrespected and abused in their work place. The majority of participants (79.6%) believed that lack of respectful care discourages pregnant women from coming to health facilities for delivery.

**Conclusions:**

The study findings indicate that most service providers from these facilities had witnessed disrespectful practices during childbirth, and recognized that such practices have negative consequences for service utilization. These findings can help decision makers plan for interventions to improve RMC taking account of the provider perspective.

## Plain English summary

Disrespect and abuse (D&A) during facility based childbirth involves provision of care that is undignified and humiliating to women. D&A is one of the main factors discouraging women from giving birth in health facilities. As an emerging area of research, standard definitions and measurement approaches to D&A are currently lacking.

This paper presents findings of a study conducted among health service providers who attend women during childbirth to assess their experiences of disrespectful and abusive practices. Fifty-seven service providers from one hospital and three health centers located in Addis Ababa, Ethiopia, completed a questionnaire. Half of the service providers reported that fellow service providers do not generally seek consent from women before performing procedures. One-quarter had witnessed fellow service providers using physical force on a woman during childbirth. Detention of women in health facilities was reported by almost one-fifth of service providers. The majority (80%) of service providers believed that D&A discourages women from giving birth in health facilities. Disrespect of the service providers themselves was also reported with more than half (57.1%) feeling that they had been disrespected and abused in their current workplace.

## Background

Maternal mortality remains a major challenge in most low and middle-income countries (LMIC) such as Ethiopia, where the maternal mortality ratio was reported to be 412 per 100,000 live births in 2015 [1, 2]. Though improving over recent years, poor utilization of maternal health services remains a problem, and undermines the country’s maternal mortality reduction goals [1, 3]. Between 2000 and 2015, maternal mortality in Ethiopia was reduced by 52.7% from 871 to 412 per 100,000 live births [1, 3]. In the same 15 year period (2000–2015), the proportion of births occurring in health facilities increased five-fold from 5% to 26% [1, 3]. While these gains are encouraging, it is still the case that a substantial majority of women are not delivering their babies in health facilities.

As revealed by several studies and national surveys, lack of courtesy and respect in health facilities and perceived poor quality of care are linked to low uptake of maternal health care services in almost all geographical regions of the country [4–11]. The presence of hostile or insensitive staff [7], disallowance of birth companions [7], disrespectful care [12], women’s lack of autonomy [6] and privacy [13], inadequate facilities in labor wards [14], and abuse by staff [14] are among the constellation of factors that actively deter women from attending for facility-based childbirth. These studies all report practices and conditions that characterize what has come to be known as disrespect and abuse (D&A), which not only discourages women from attending for facility-based deliveries but also denies their rights to high quality childbirth services as declared by the United Nations [15, 16].

To date, there is no standardized definition of D&A during childbirth, also known as ‘mistreatment’ or ‘obstetric violence’. Freedman and colleagues proposed a preliminary definition as “interactions or facility conditions that local consensus deems to be humiliating or undignified, and those interactions or conditions that are experienced as or intended to be humiliating or undignified” [17]. Triggered by the high levels and severity of D&A reported across the world, in 2014 the World Health Organization drafted and endorsed a statement that aims to prevent and eliminate D&A during childbirth globally by advancing respectful maternity care (RMC) [18]. RMC is defined as “the humane and dignified treatment of a childbearing woman throughout her pregnancy, birth, and the period following childbirth” [19] and embodies: respecting woman’s choices and rights, treating her with reverence, and manifesting supportive communications and actions [19–22]. In response, there have been moves to draft and adopt a consensual definition of D&A [17], establish more comprehensive typologies of D&A [23], and develop standardized measurement tools [24].

A landscape analysis undertaken by Bowser and Hill (2010), established a framework depicting factors contributing to D&A [16]. In 2015, Bohren and colleagues conducted a mixed-method systematic review to further categorize D&A [23]. Seven categories of D&A were generated: physical abuse, verbal abuse, sexual abuse, stigma and discrimination, failure to meet professional standards of care, poor rapport between women and providers, and health system conditions and constraints. Although several qualitative studies describing mistreatment during childbirth have been conducted [16, 23, 25–29], there are only a small number of prevalence studies estimating the magnitude of D&A [26–30]. However, the methodological approaches taken in these studies are inconsistent, in part because there is no universally agreed definition of D&A and no internationally recognized approach to D&A measurement. These inconsistencies make it difficult to make comparisons between settings and within settings over time. Additionally, most quantitative assessment of D&A in health facilities has been captured from the client perspective, not the provider perspective, which is a major gap in the literature. Capturing health care providers’ practices in relation to D&A during childbirth will strengthen the available evidence. The provider perspective is essential for the development of meaningful definitions of D&A, accurate measurement tools, and effective interventions. Therefore, this study aims to assess service providers’ experiences of D&A of women during facility-based childbirth.

## Methods

### Study design and setting

The study reported in this paper is based on analysis of data collected as a component of a larger quantitative study, which was conducted to estimate the prevalence of D&A during facility-based childbirth in Addis Ababa in August 2013 [27]. Three health centers and one teaching hospital were included in the study. According to the three tier Ethiopian health care system, health centers are categorized in the first tier i.e. as primary health care units that refer clients to primary hospitals. The study hospital is a specialized teaching hospital which serves a population of five million.

### Population of the study and sample size

Participants in this study were health professionals (midwives, clinical nurses, health officers, and medical doctors), all of whom assisted women at the time of childbirth during the study period (August to September, 2013). A minimum service of six consecutive months in the labour room prior to the data collection period was a criterion for inclusion in the study. This ensured enough time for familiarization with the context and work culture. The study aim was to generate evidence on service providers’ experiences of D&A during childbirth. We included all eligible service providers who were willing and available to participate from all four institutions. Accordingly, all service providers (*N* = 61) who were on duty in the labor wards during the study period were invited for inclusion in the study. Four service providers did not complete the questionnaire making the final number of participants 57; 34 from the hospital and 23 from the health centers.

### Research instrument, data collection and processing

The data collection tool was a self-administered questionnaire comprised of four categories: socio-demographic characteristics; professional and work-related characteristics; attitudes and practices related to RMC; and recognition of mistreatment of women during childbirth. A performance standard prepared by the Maternal and Child Health Integrated Program [31] was adapted and translated into local language (Amharic) to assess service providers experiences on D&A. The tool incorporated all seven major types of D&A suggested by Bowser and Hill (physical abuse, non-consented care, non-confidential care, non-dignified care, discrimination, denying care or abandonment, and detention in facilities) [16]. The section assessing service providers’ practices related to D&A in light of RMC included 14 questions with possible responses of either “Always”, “Sometimes” or “Never”. Eight questions asked about service providers’ experiences of witnessing D&A during facility-based childbirth, categorized into “Ever in the past” or “Within the past 30 days”. One additional question was included to identify the proportion of respondents who admitted to disrespecting a client, “In your own personal capacity have you ever done anything that made you feel you disrespected or abused women in childbirth?” Another question asked service providers if they had ever been disrespected in their work place by patients, other staff, or health facility administration.

The original questionnaire was developed in English, and later translated into Amharic, the official working language in health facilities. The principal investigator invited potential participants to be part of the study and provided information on the study and how to complete the questionnaire. This took place during group information sessions at the study hospital, and individually at the health centers. To maintain anonymity, completed questionnaires were placed into a sealable envelope by participants, and subsequently collected by a data collector.

SPSS version 16 software was used to enter, clean, and analyze data. Descriptive statistics were used to display frequencies and proportions for all variables. Additionally, the proportion of respondents who had seen at least one episode of D&A during childbirth in their health facility was calculated.

### Data quality management

The questionnaire was pretested in a similar health center that was not included in the study. Five midwives and a clinical nurse completed the questionnaire during the pilot phase. Following this, modifications were made to the questionnaire, especially in relation to the translation of “disrespect and abuse” into the local language, and changing the questions into third person.

## Results

### Socio-demographic and service related characteristics of service providers

A total of 57 health professionals agreed to participate in the survey. Just under 60% were from the hospital while the rest were from the three participating health centers in Addis Ababa (Table 1).

**Table 1:** Socio-demographic and service related characteristics of service providers, Addis Ababa, 2013

Characteristics	Frequency (%)
Type of respondents’ health facility	
Hospital	34 (59.6)
Health center	24 (40.4)
Total (N)	57 (100.0)
Sex	
Male	20 (35.1)
Female	37 (64.9)
Total (N)	57 (100.0)
Age in years (*n* = 49)	
29 years and below	41 (83.7)
30 years and above	8 (16.3)
Total (n)	49 (100.0)
Mean ± SD = 27.25 ± 5.45 years	
Current Profession	
Clinical Nurse	13 (22.8)
Midwife	25 (43.9)
Medical Doctor	16 (28.1)
Health Officer	2 (3.5)
Gynecologist/Obstetrician	1 (1.8)
Total (N)	57 (100.0)
Service year with the current profession (in years)	
≤ 5	44 (77.2)
6–10	6 (10.5)
≥ 11	7 (12.3)
Total (N)	57 (100.0)
Median ± IQR = 3 ± 3	
Estimated service hours per day in MCH settings (*n* = 54)	
< 9 h	39 (22.5)
9–12 h	81 (46.8)
> 12 h	53 (30.6)
Total (n)	54 (100.0)
Mean ± SD = 9.64 ± 2.44 h	
Estimated number of deliveries service providers attend per day (*n* = 32)	
≤ 3	17 (53.1)
≥ 4	15 (46.9)
Total (n)	32 (100.0)
Mean ± SD = 3.56 ± 2.0	

The mean ± SD age of the participants was 27.25 ± 5.45 years, and the majority (83.7%) were below the age of 30 years. Two thirds (64.9%) were female, 25 (43.9%) were midwives, 13 (22.8%) were clinical nurses, 16 (28.1%) were medical doctors and one was an obstetrician. The majority (77.2%) had less than 5 years’ experience. The mean ± SD duration of working hours per day was 9.64 ± 2.44 and they attended an average of 3.56 ± 2.0 deliveries per day (Table 1).

### Service providers’ perceptions of their work environment

The majority of participants felt that they had a high (52.6%) or very high (31.6%) work load at their facility. Forty percent felt that they received either poor or very poor support from their facility management, and only 29.8% felt comfortable with the working environment at their facility, meaning they were comfortable with workplace harmony and management support. Despite this, 75.5% of respondents said they enjoyed providing childbirth health services, 87.7% were satisfied with their work, and 58.2% want to continue working in their facility (Table 2).

**Table 2:** Service providers’ perceptions of their work environment, Addis Ababa, 2013

Characteristics	Frequency (%)
How do you rate your work load in your facility?	
Very high	18 (31.6)
High	30 (52.6)
Medium	9 (15.8)
Low	–
Very low	–
Total (N)	57 (100.0)
How do you rate the management support you receive from your health facility?	
Very Good	8 (14.5)
Good	15 (27.3)
Medium	10 (18.2)
Poor	9 (16.4)
Very Poor	13 (23.6)
Total (n)	55 (100.0)
How comfortable^*^ is the work environment in your health facility?	
Very Good	8 (14.0)
Good	9 (15.8)
Medium	24 (42.1)
Poor	8 (14.0)
Very Poor	8 (14.0)
Total (N)	57 (100.0)
How happy are you providing childbirth services?	
Very Happy	31 (54.4)
Happy	12 (21.1)
Moderately Happy	10 (17.5)
Unhappy	2 (3.5)
Very Unhappy	2 (3.5)
Total (N)	57 (100.0)
How satisfied are you with your work?	
Very Satisfied	22 (38.6)
Satisfied	28 (49.1)
Neutral	6 (10.5)
Unsatisfied	–
Very Unsatisfied	1 (1.8)
Total (N)	57 (100.0)
Do you want to work for your facility in the future? (*n* = 55)	
Yes	32 (58.2)
No	23 (41.8)
Total (n)	55 (100.0)

^*^harmony and facilitation (management and supervision) at work place

### Service providers’ observations of disrespect and abuse during facility-based childbirth using a respectful maternity care framework

Service providers assessed the provision of respectful care in facility-based childbirth in their health facilities by rating fourteen Likert scale question with three staged responses (see Table 3). Low levels (meaning ≤50%) of several respectful behaviors were consistently observed: Specifically, providers always introducing themselves to laboring mothers (8.8% always, 40.4% never); allowing women to assume the position of their choice during birth (20.4% always, 20.4% never); encouraging the woman’s companion to remain with her (24.6% always, 40.4% never); providing appropriate pain relief (41.1% always, 19.6% never); encouraging mothers to ask questions during labor (42.1% always, 17.5% never); obtaining consent prior to procedures (47.4% always, 14% never).

**Table 3:** Service providers’ experiences of disrespect and abuse using a respectful maternity care framework, Addis Ababa, 2013

Respectful maternity care elements	Response, *n* (%)		
	Always	Sometimes	Never
Do service providers, in this facility			
Provide appropriate pain relief or comfort measures for laboring mothers? (*n* = 56)	23 (41.1)	22 (39.3)	11 (19.6)
Introduce themselves to laboring mothers? (*n* = 57)	5 (8.8)	29 (50.9)	23 (40.4)
Encourage a mother’s companion to remain with her, whenever possible? (*n* = 57)	14 (24.6)	20 (35.1)	23 (40.4)
Convey information to mothers at a language-level they can understand? (*n* = 56)	32 (57.1)	24 (42.9)	–
Encourage mothers to ask questions during labor? (*n* = 57)	24 (42.1)	23 (40.4)	10 (17.5)
Respond to mothers’ questions with promptness, politeness, and truthfulness? (*n* = 57)	36 (63.2)	20 (35.1)	1 (1.8)
Explain to mothers what is being done and what to expect throughout labor and birth? (*n* = 56)	33 (58.9)	23 (41.1)	–
Provide periodic updates on status and progress of labor to laboring mothers? (*n* = 56)	28 (50.0)	24 (42.1)	4 (7.1)
Allow mothers to move around during labor? (*n* = 57)	32 (56.1)	19 (33.3)	6 (10.5)
Allow mothers to assume the position of her choice during birth? (*n* = 54)	11 (20.4)	32 (59.3)	11 (20.4)
Obtain consent or permission of mothers prior to any procedure? (*n* = 57)	27 (47.4)	22 (38.6)	8 (14.0)
Use curtains or other visual barriers to protect mothers during exams, births and procedures? (*n* = 55)	33 (60.0)	15 (27.3)	7 (12.7)
Are mothers encouraged to call for help if they are in need? (*n* = 55)	33 (60.0)	15 (27.3)	7 (12.7)
Is it easy for service providers to respond to mothers’ calls for help? (*n* = 57)	44 (77.2)	10 (17.5)	3 (5.3)

### Service providers’ personal observations of disrespectful and abusive care during facility-based childbirth

A quarter of the respondents (25.9%) reported ever witnessing use of physical force or abrasive behavior such as staff slapping or hitting laboring women. Only 4 (7.4%) had observed mothers being separated from their baby unnecessarily, and only 7 (13.2%) had seen mothers left alone or unattended during labor. A third of participants (34.5%) reported that mothers’ privacy during labor and delivery was not always protected. Detaining mothers at the facility, against their will, was observed by 9 (18%) participants. Eight participants (14.5%) reported (ever) having personally done things that they feel is disrespectful and abusive to women (Table 4). Ensuring women’s privacy during childbirth was also a prevailing problem in the past 30 days before the survey; 35.6% of participants had seen mothers whose privacy was not protected. Two of the participants have also reported they have done things that they feel is disrespectful to women in the last 30 days (Table 4).

**Table 4:** Service providers’ personal observations of disrespectful and abusive care during childbirth in health facilities, Addis Ababa, 2013

Questions related to disrespect and abuse	Response; Yes, *n* (%)	
	Ever past (n1)^a^	Within the past 30 days (n2)^b^
Physical force or abrasive behavior with laboring mothers (for example slapping or hitting them) (n1 = 54; n2 = 44)	14 (25.9)	1 (2.3)
Mothers have been separated from their baby unnecessarily (n1 = 54; n2 = 45)	4 (7.4)	3 (6.7)
Mothers have been denied foods or fluids unnecessarily (n1 = 55; n2 = 45)	5 (9.1)	2 (4.4)
Service providers have used insults, intimidation, threats, or coercion to mothers or their companion (n1 = 53; n2 = 45)	9 (17.0)	4 (8.9)
Service providers shown disrespect to mothers based on any specific attribute. (HIV status or ethnic group, for example) (n1 = 52; n2 = 44)	4 (7.7)	–
Mothers have been left alone or unattended (n1 = 53; n2 = 45)	7 (13.2)	4 (8.9)
Mothers’ privacy during labor and delivery has not been protected (n1 = 55; n2 = 45)	19 (34.5)	16 (35.6)
Mothers have been detained at the facility, against their will (n1 = 50; n2 = 41)	9 (18.0)	2 (4.9)
In your own personal capacity have you ever done anything that made you feel you disrespected or abused women in childbirth? (n1 = 55; n2 = 45)	8 (14.5)	2 (4.4)

^a^n1: number of respondents for respective questions aimed at practice of disrespect and abuse ever past in the facility

^b^n2: number of respondents for respective questions aimed at practice of disrespect and abuse within the last 30 days of the interview

### Service providers’ perceptions of the consequences of disrespectful and abusive care during childbirth

More than half (57.1%) of participants felt that they themselves had been disrespected or abused in their work place (by clients or health providers). The majority (79.6%) believed that lack of respectful care discourages pregnant women from coming to health facilities for delivery (Table 5).

**Table 5:** Service providers’ perception on effect of disrespectful care towards utilization of skilled care at birth, Addis Ababa, 2013

Questions related to disrespect and abuse	Response; Yes, n (%)	
	Yes	No
Do you think you have ever been disrespected or abused at your work place by anyone? (*n* = 56)	32 (57.1)	24 (42.9)
Do you think lack of respectful care is a factor which discourages pregnant women from coming to health facilities for delivery? (*n* = 54)	43 (79.6)	11 (20.4)

## Discussion

This study reports on service providers’ experience of D&A using data from a parallel study which was conducted in Addis Ababa, in 2013 [27]. The previous study reported on D&A from the clients’ perspective using exit interviews of women who delivered in the study facilities. The current study provides evidence of D&A from the perspective of service providers. Comparing the level of D&A from the two perspectives is not possible; women report individual experiences of D&A while multiple providers may witness D&A of one woman.

Close to 80% of participants in the current study believed that lack of respectful care discourages women from having facility based childbirth. Furthermore, the study participants’ observations indicate major gaps in the practice of RMC, although this does not mean they are knowledgeable about RMC. Service providers’ poor attitudes towards RMC have been reported as a reason for non-adherence to recommended RMC practices [32–34]. In this regard, improving service providers’ knowledge of RMC has proven to be a successful intervention in Kenya [35], and Tanzania [36].

A high proportion of participants in the current study had not only witnessed practices of D&A during childbirth but also reported feeling disrespected in their work place, either by clients or other facility staff. This widespread observation and experience of D&A suggests it is normalized in the culture of the study health facilities. Normalization of D&A has been identified as one of the main risk factors for D&A [16, 37].

In this study, questions were asked in the “third person” format to minimize chance of social desirability bias. However, residual social-desirability factors may still be influencing the participants’ responses (Eg. Pride in the facility). The level of detention reported in the current study (18%, ever) might not indicate the current situation. This can be explained by the fact that participants who have been working since before the introduction of user fee exemption (2005–2010) for maternal health services had observed detention of women.

High work load (83.2%), poor support from facility management (40%), and the discomfort of the work environment (28%) revealed by this study necessitates a multidimensional effort to improve the quality of childbirth services in these settings. A systematic review of the impacts of facility level interventions on the quality of maternal and newborn health care identified that facility level management interventions and stress management bring positive change to job satisfaction and an overall improvement of desired practices [38]. A process evaluation from Benin demonstrated that introduction of RMC interventions resulted in improvement of midwives treatment of women during childbirth when leadership and commitment of hospital management to RMC is evident [39]. However, it must be noted that one-off interventions, such as short term trainings and workshops, will not bring about sustainable change [40]. Designing interventions to bring about sustainable positive changes in RMC need to incorporate systems level thinking and action [40].

A high level (57%) of participants in this study said they had been disrespected and abused in their work place (by clients or health providers). However, the sample size is too small to meaningfully test the relationship between service providers’ experience of disrespect and abuse and witnessing or perpetrating D&A. A disrespectful culture in the health facility is believed to be one of the factors contributing to D&A [41]. The possibility of intersectionality between workplace culture and environment and service providers’ practices and attitudes towards women during childbirth is worthy of future investigation.

This study is among a very few studies conducted in LMICs to generate quantitative evidence of disrespectful and abusive practices during facility based childbirth from the service provider perspective. However, the study is limited in terms of reporting an association between key variables due to the small sample size. Additionally, the lack of accepted D&A measurement indicators may have compromised the validity of the data collection instrument.

## Conclusions

Service providers who attend childbirth in Addis Ababa witness disrespectful and abusive treatment of women by fellow professionals and also themselves can feel disrespected and abused in their work place. Addressing supply side barriers to the provision of RMC needs to focus on improving work performance of service providers by not only providing them with context-specific training on RMC, but also instilling a system-wide culture of respect in health facilities. This will have the effect of protecting the rights of both women and service providers.

## Abbreviations

D&A: Disrespect and Abuse
IQR: Interquartile Range
LMIC: Low and Middle-Income Countries
RMC: Respectful Maternity Care
SPSS: Statistical Package for the Social Sciences
